# MicroRNA expression profiles from eggs of different qualities associated with post-ovulatory ageing in rainbow trout (*Oncorhynchus mykiss*)

**DOI:** 10.1186/s12864-015-1400-0

**Published:** 2015-03-17

**Authors:** Hao Ma, Gregory M Weber, Mark A Hostuttler, Hairong Wei, Lei Wang, Jianbo Yao

**Affiliations:** Division of Animal and Nutritional Sciences, West Virginia University, Morgantown, WV 26506 USA; National Center for Cool and Cold Water Aquaculture, USDA/ARS, Kearneysville, WV 25430 USA; School of Forest Resources and Environmental Science, Michigan Technological University, Houghton, MI 49931 USA

**Keywords:** microRNA, Egg quality, Post ovulation, Rainbow trout

## Abstract

**Background:**

Egg quality is an important aspect in rainbow trout farming. Post-ovulatory aging is one of the most important factors affecting egg quality. MicroRNAs (miRNAs) are the major regulators in various biological processes and their expression profiles could serve as reliable biomarkers for various pathological and physiological conditions. The objective of this study was to identify miRNAs that are associated with egg qualities in rainbow trout using post-ovulatory aged eggs.

**Results:**

Egg samples from females on day 1, day 7, and day 14 post-ovulation (D1PO, D7PO and D14PO), which had the fertilization rates of 91.8%, 73.4% and less than 50%, respectively, were collected and small RNAs isolated from these samples were subjected to deep sequencing using the Illumina platform. The massive sequencing produced 27,342,477, 26,910,438 and 29,185,371 reads from the libraries of D1PO, D7PO and D14PO eggs, respectively. A three-way comparison of the miRNAs indicated that the egg samples shared 392 known and 236 novel miRNAs, and a total of 414, 481, and 470 known and 243, 298, and 296 novel miRNAs were identified from D1PO, D7PO and D14PO eggs, respectively. Four known miRNAs (omy-miR-193b-3p, omy-miR-203c-3p, omy-miR-499-5p and omy-miR-7550-3p) and two novel miRNAs (omy-miR-nov-95-5p and omy-miR-nov-112-5p) showed significantly higher expression in D1PO eggs relative to D14PO eggs as revealed by both deep sequencing and real time quantitative PCR analysis. GO analysis of the predicted target genes of these differentially expressed miRNAs revealed significantly enriched GO terms that are related to stress response, cell death, DNA damage, ATP generation, signal transduction and transcription regulation.

**Conclusions:**

Results indicate that post-ovulatory ageing affects miRNA expression profiles in rainbow trout eggs, which can in turn impact egg quality. Further characterization of the differentially expressed miRNAs and their target genes may provide valuable information on the role of these miRNAs in controlling egg quality, and ultimately lead to the development of biomarkers for prediction of egg quality in rainbow trout.

**Electronic supplementary material:**

The online version of this article (doi:10.1186/s12864-015-1400-0) contains supplementary material, which is available to authorized users.

## Background

Fish egg quality is defined as the capability of an egg to become fertilized and subsequently develop into a normal embryo or the probability of eggs to exhibit low mortalities at fertilization, eyeing, hatching, and first feeding [1]. The production of high quality eggs is a major objective of the aquaculture industry, as egg quality not only affects fertilization rate, but also is an important attribute of robust embryonic development [2,3]. However, visible differences between good and bad eggs at oviposition is not usually conspicuous in rainbow trout, and therefore, the inclusion of eggs from individual females with poor egg quality into mass incubation units not only results in unexpected losses in egg production, but also problems associated with the removal of dead eggs and embryos after fertilization and fungi infection in the hatchery [4]. Therefore, enabling evaluation of the egg quality before fertilization is highly desirable in aquaculture production.

In teleost fish, a mature egg is developed through multiple phases, including primary oocyte growth, secondary growth including the cortical alveolus stage and vitellogenesis, follicle maturation and ovulation [5,6]. The coordinated multiple developmental stages can be affected by many genetic, biological, and environmental factors [3]. It has been reported that the quality of rainbow trout eggs is dependent not only on the genetic characteristics of parents [5], but also the age of female [7], and are susceptible to environmental influences, such as the diet of brood fish [8-12], stress [13-15], photoperiod [16], and the physiochemical conditions of the water [17]. All of these factors make egg quality highly variable and difficult to control [18,19]. As the ovulated eggs in reared rainbow trout do not usually oviposit naturally, post-ovulatory aging of the eggs is widely accepted as a common determinant for egg quality [20-22].

The importance to distinguish good and bad quality eggs before fertilization has driven studies on the identification of markers associated with egg quality in rainbow trout. Wojtczak and coworkers found that very poor quality eggs turn water turbid [23]. Egg survival rate has also been associated with aspects of egg composition [20]. The total amount of water imbibed after 30 minute incubation has been recognized as an indicator of egg quality [24]. In addition, molecular markers that are potential indicators of egg quality have also been reported. Such markers include the maternally-derived IGF-I and IGF-II [25], and LVII fragments as well as other proteins identified from the coelomic fluid [26]. Many specific genes that are potentially involved in the regulation of oocyte maturation, egg developmental potential, and embryo survival were identified by microarray and quantitative real time PCR (RT-qPCR) analyses [3,27,28]. Although the above studies have attempted to address the critical factors responsible for the observed variability of egg quality, the molecular mechanisms underpinning the regulation of egg quality in rainbow trout remain largely elusive.

Recent advances in epigenetic research have demonstrated that the evolutionarily conserved microRNAs (miRNAs) play important roles in differentiation and maturation of various cell types [29,30]. In zebrafish, mutant embryos lacking mature miRNAs had severe deformity during embryogenesis [31-34]. In mouse, although the miRNA functions are suppressed during oocyte maturation, the maternal miRNAs are critical to normal embryonic development [35-38]. In addition, many miRNAs have been shown to play roles in programmed cell death [38-42]. A comprehensive review of miRNAs in teleost fish development, reproduction and response to environmental stimuli was published recently [43]. In order to identify the miRNAs that might play important roles in oocyte and embryonic development in rainbow trout, the expression of miRNAs in rainbow trout eggs and early embryos have been studied and some novel egg-predominant miRNAs were identified [44,45]. In this study, post-ovulatory aged rainbow trout eggs with different qualities were collected and used to generate miRNA transcriptome profiles for identifying specific miRNAs associated with egg quality, which could potentially be used as biomarkers for evaluating egg quality. The study also provides new information and insights for future studies to elucidate the gene regulatory networks involved in the control of egg quality.

## Results

### Identification of known and novel miRNAs in eggs of different qualities

Small RNA libraries constructed from eggs of different ages post-ovulation were subjected to deep sequencing using the Illumina platform. The massive sequencing produced 27,342,477, 26,910,438 and 29,185,371 reads from the libraries constructed from D1PO, D7PO and D14PO eggs, respectively (Table 1). After removing the impurity sequences, known mRNAs, non-coding RNA families without miRNA, and repetitive DNA sequences, the remaining 23,554,621, 26,144,764 and 28,408,709 sequences from these three samples were used for identification of known miRNAs and prediction of new miRNAs.

**Table 1:** **Number of sequences generated from small RNA libraries of eggs with different qualities**

**Items**	**D1PO**	**D7PO**	**D14PO**
Total reads	27,342,477	26,910,438	29,185,371
Mappable reads	23,554,621	26,144,764	28,408,709
Reads mapped to miRbase	2,945,228	2,261,910	1,464,754
Reads for novel miRNA prediction	13,421,585	14,105,022	15,709,247

A total of 2,945,228, 2,261,910 and 1,464,754 sequences from D1PO, D7PO and D14PO eggs, respectively, were mapped to miRbase database (Release 21). Based on the criteria described in “Methods” section for known miRNAs, a total of 496 known miRNAs were identified from the three samples (Additional file 1: Table S1). Predication of novel miRNAs was carried out according to the criteria that the extended sequences of the miRNAs at the aligned rainbow trout genomic locations have the propensity of forming hairpin structures, and the sequences do not meet the criteria of known miRNAs. The number of novel miRNAs predicted from our datasets was 306 (Additional file 1: Table S2).

### Identification of differentially expressed miRNAs in eggs of different qualities

**Figure 1:**
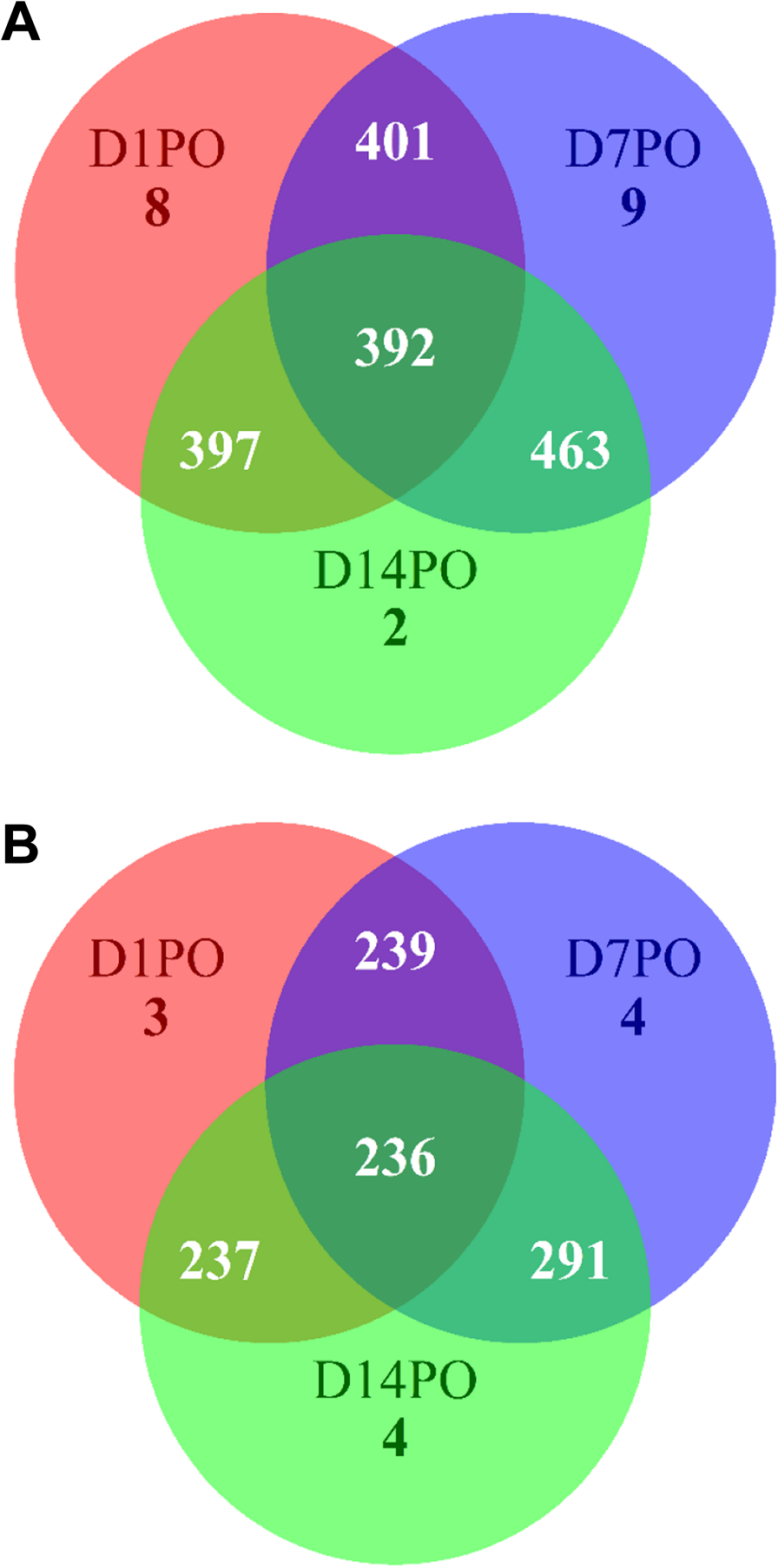
**Three way Venn diagrams showing the number of miRNAs among D1PO, D7PO and D14PO eggs. (A)**. Known miRNAs identified. **(B)**. Novel miRNAs predicted. D1PO, D7PO, and D14PO are day 1, 7, and 14 post-ovulatory eggs, respectively.

**Figure 2:**
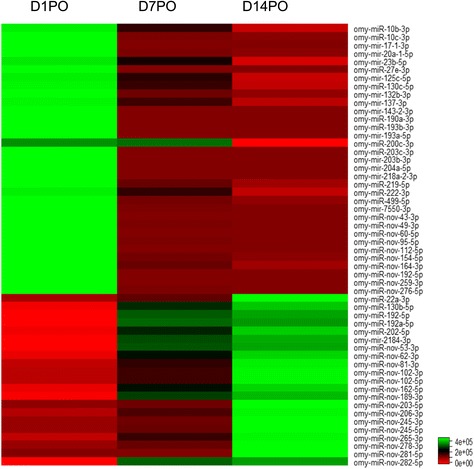
**Expression profiles of miRNAs in eggs of different qualities.** The miRNAs with more than 10 times difference in expression between D1PO and D14PO eggs are shown.

**Figure 3:**
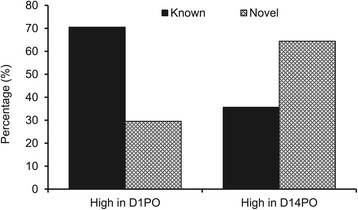
**Percentage of known and novel miRNAs showing higher expression in high quality eggs (D1PO) or low quality eggs (D14PO).**

A three-way comparison of the miRNAs among the samples indicated that D1PO, D7PO and D14PO eggs shared 392 known and 236 novel miRNAs (Figure 1). A total of 414, 481, and 470 known miRNAs and 243, 298, and 296 novel miRNAs were identified from D1PO, D7PO, and D14PO samples, respectively (Table 2). To identify miRNAs that are related to egg quality, the miRNA reads in D1PO and D14PO samples were quantile normalized and compared. A total of 189 miRNAs showed differential expression between the 2 samples (fold change greater than 3). Eighty-eight miRNAs showed higher expression in high quality eggs, while 101 miRNAs displayed higher expression in low quality eggs (Additional file 1: Table S3). Differentially expressed miRNAs with a fold change greater than 10 are shown in Figure 2. Interestingly, the majority of the miRNAs highly expressed in D1PO eggs are known miRNAs (70.45%), while majority of the miRNAs with higher expression in D14PO eggs are novel miRNAs (64.36%) (Figure 3).

**Table 2:** **Number of known and novel miRNAs identified from eggs of different qualities**

**miRNAs**	**D1PO**	**D7PO**	**D14PO**
Known (total)	414	481	470
Novel (total)	243	298	296
Known (specific)	8	9	2
Novel (specific)	3	4	4

**Figure 4:**
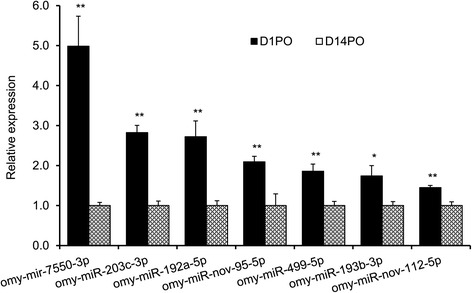
**RT-qPCR validation of differentially expressed miRNAs identified by deep sequencing between D1PO and D14PO eggs.** Data were normalized using β-actin and 18S rRNA. The means of the normalized miRNA expression values (n = 4 pools) were calculated and expressed as relative fold changes. Only omy-miR-192a-5p does not match the sequencing data.

The differentially expressed miRNAs with a fold change greater than 10 and normalized reads greater than 50 in both samples were subjected to real-time quantitative PCR (RT-qPCR) analysis. Based on melting curve analysis, we selected 7 miRNAs that showed specific amplifications for further analysis (Additional file 2: Figure S1). Six of the 7 miRNAs that were successfully analyzed by RT-qPCR showed significantly higher expression in D1PO vs. D14PO eggs, which is consistent with the deep sequencing results, although the magnitude of fold changes shown by the two methods was not the same (Figure 4). Of the 6 miRNAs, 4 are known miRNAs (omy-miR-193b-3p, omy-miR-203c-3p, omy-miR-499-5p and omy-miR-7550-3p) and 2 are novel miRNAs (omy-miR-nov-95-5p, omy-miR-nov-112-5p).

### Analysis of predicted targets of the differentially expressed miRNAs

**Figure 5:**
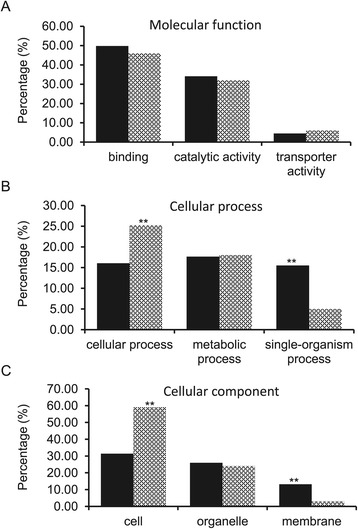
**Top 3 GO terms (second level) of the target genes of 6 miRNAs highly expressed in high quality eggs (D1PO) compared with the same GO terms of whole transcriptome in rainbow trout. (A)** Molecular function. **(B)** Biological process. **(C)** Cellular component. Solid fill bar: Top 3 GO terms of the target genes of 6 differentially expressed miRNAs; Pattern fill bar: the corresponding GO terms analyzed using whole genome transcriptome. ** indicates significant difference at P < 0.001.

PITA and miRanda algorithms were used to predict the target genes of the 6 differentially expressed miRNAs that were validated by RT-qPCR analysis, A total of 178 gene entries from gene index database (http://www.animalgenome.org/repository) were predicted, which represent 114 known genes and 23 unknown genes (Additional file 1: Table S3). In addition, when mitochondrial genome was used as a query, a gene encoding cytochrome c oxidase subunit 1 (*COX6B1*) was predicted as the target of omy-miR-nov-95-5p. GO functional enrichment analysis of the target genes was carried out using Blast2GO software [46]. The results indicated that the top three GO terms (second level) in biological process include cellular process, metabolic process, and single organismal process, and the most significant GO terms in molecular function are binding, catalytic activity, and transporter activity (Figure 5). In comparison with the recent transcriptome data in rainbow trout [47], the significantly enriched GO terms are single-organism process and membrane. Interestingly, the GO term of cell death, which is one of the indicators of egg quality, is under the children branches of single-organism process.

GO enrichment analysis using 96,546 rainbow trout unique transcript sequences as the background showed that 23, 23, and 90 GO terms in cellular component, molecular function, and biological process, respectively, are significantly enriched (Adjusted p-value < 0.05) (Additional file 1: Table S5). The genes associated with the significantly enriched GO terms are mainly involved in response to stress and DNA/RNA damage (*RPB4*, *RECQ4A*, *CHD1L*, *WDR61*, and *CNOT1*), cell death and signal transduction (*RASSF5*, *GEM*, *RAB14*, and *CACNA1E*), energy and transcription regulation (*ATP5A1*, *COX6B1*, *CDT1*, *GTF2A2*, *SIX1*, and *GMEB2*).

## Discussion

Using deep sequencing in combination with RT-qPCR, we have identified 6 miRNAs that are associated with egg quality in rainbow trout. These miRNAs could potentially be used as biomarkers for prediction of egg quality in rainbow trout. Our results showed that the numbers of known and novel miRNAs do not show dramatic changes among eggs of different qualities (Table 2), however, most of the highly expressed miRNAs in high quality eggs are known miRNAs and most of the highly expressed miRNAs in low quality eggs are novel miRNAs.

In the study, we used post ovulatory aged eggs with different fertilization rates to identify miRNAs that are associated with egg quality. The D1PO, D7PO and D14PO eggs contained both “good” and “bad” eggs of varying proportions resulting from temporal change of eggs held in the body cavity. Therefore, the miRNAs among these egg samples have only quantitative discrepancy. It is conceivable that identification of evident miRNA expression discrepancy among these samples with varying proportion of “good” and “bad” eggs is challenging, and such difficulties have also been documented in previous studies [25,48]. The SYBR green based RT-qPCR method for miRNA detection has its limitations. As both the universal primer and the miRNA specific primer are fixed, it makes optimization of the assay very difficult. Only 7 miRNAs showed specific amplification based on melting curve analysis and many more did not show specific amplifications, although different annealing temperatures were tried.

Mitochondrion is not only vital in ATP generation and maintenance of cell homeostasis, but also central in the apoptotic signaling pathways [49]. It has been reported that some miRNAs were involved in the regulation of mitochondrion-mediated apoptosis [50,51]. In this study, one of the differentially expressed novel miRNAs, omy-miR-nov-95-5p, was predicted to target mitochondrial gene (*COX6B1*). *COX6B1* is known to catalyze the electron transfer from reduced cytochrome c to oxygen in respiratory chain, and deficiency of cytochrome c oxidase is linked to many human diseases [52]. Therefore, down-regulated expression of omy-miR-nov-95-5p in aged eggs may cause abnormal expression of *COX6B1*, thereby affecting normal mitochondrial respiratory chain and egg quality. In addition, many predicted target genes of omy-miR-nov-95-5p and omy-miR-193b-5p are associated with significantly enriched GO terms, which include cell death, stress response, DNA damage and repair, and RNA degradation. These genes include *RASSF5*, *RPB4*, *RECQ4A*, *CHD1L*, *WDR61*, and *CNOT1*. Abnormal expression of these important genes may also contribute to decreased quality of D14PO eggs. Some of the differentially expressed miRNAs identified in this study, such as miR-449 and miR-203, have been reported to affect cell death and tumor suppression [53,54], but the other differentially expressed miRNAs have not been characterized with regard to their functions. Therefore, a comprehensive study of these miRNAs and their target genes would help understanding the factors contributing to egg quality.

Many factors can affect miRNA expression [41,55]. In rainbow trout, the eggs kept in the cavity for extended time are associated with reduced levels of *IGF I* and *IGF II*, and increased levels of *KRT8*, *CTSZ* and other transcripts [25,48]. Increased activities of *GOT1*, *ACPP*, *LVII* fragments and others biomolecules in the coelomic fluid have also been shown to be related to egg quality [22,26]. In addition, the levels of 17α 20β-P and 17α-OH-P in blood vary significantly before and after ovulation [21]. It is not known if these changes may directly or indirectly affect miRNA expression, leading to the changes in target gene expression. Furthermore, some miRNAs have regulatory roles in controlling other miRNAs [56]. Therefore, in order to understand the mechanisms underlying the changes in egg quality such as those associated with post-ovulation aging in rainbow trout, it would be important to systematically study the interaction networks among physiological and environmental factors affecting egg quality, the miRNAs and their target genes.

## Conclusions

This study identified 6 differentially expressed miRNAs that are associated with egg quality in rainbow trout. Further characterization of these miRNAs, especially the novel ones, and their target genes may provide valuable information on the roles of these miRNAs in controlling egg quality, and ultimately lead to the development of novel biomarkers for evaluation of egg quality in rainbow trout.

## Methods

### Ethics statement

All experiments were conducted under a protocol approved by the USDA/ARS National Center for Cool and Cold Water Aquaculture Institutional Animal Care and Use Committee (protocol #70).

### Sample collection and determination of fertilization rate

Samples from commercial populations of Troutlodge Inc. (Sumner, WA) were reared for at least two generations using commercial trout feed under the temperature of 12.0-12.5°C in treated spring water recirculating partially in the National Center for Cool and Cold Water Aquaculture (Kearneysville, WV). The fish were checked daily for ovulation as described previously [57]. Eggs were collected from 32 females on day 1, day 7, and day 14 post-ovulation (D1PO, D7PO, and D14PO) as described previously [57,58]. The eggs for RNA isolation were placed in microcentrifuge tubes and frozen in liquid nitrogen after removal of coelomic fluid, and then stored in −80°C freezer until extraction of RNA.

Eggs fertilized using pooled milt from at least three males were placed in Davidson’s solution for microscopic analysis to determine fertilization rate. Fertilization rate was based on cleavage evaluation of 50 embryos per group as described previously [57,58]. The fertilization rates for D1PO, D7PO, and D14PO were 91.8%, 73.4% and less than 50%, respectively.

### Sequencing and analysis of egg miRNAs

Total RNA from the eggs was isolated using Trizol reagent (Invitrogen, Carlsbad, CA) according to the manufacturer’s instructions followed by additional purification steps with lithium chloride precipitation. The RNA integrity was evaluated by gel electrophoresis, and the RNA purity was checked by the ratio of OD_260_/OD_280_. The RNAs isolated from eggs of different females were pooled, and the pooled RNAs were used for sequencing of miRNAs, which was performed on an Illumina GAIIx by LC Sciences (Huston, TX) as described previously [45]. The software package, ACGT101-miR v3.5 from LC Sciences (Houston, TX), was used for analyzing the sequencing data. Sequences with low resolution, copy number less than 10, length shorter than 15 nt or longer than 26 nt, adapter sequences, junk sequences, and simple sequence were filtered out. In addition, the sequences mapped to the databases of mRNA (ftp://ftp.ncbi.nih.gov/genomes/D_rerio/RNA/Gnomon_mRNA.fsa.gz), Rfam (http://rfam.janelia.org) and Repbase (http://www.girinst.org/repbase) were also removed. The remaining sequences were used to BLAST against all miRNAs in the miRBase database (release 21) to identify known miRNAs (less than 2 mismatches in the first 18 nt or E-values equal or smaller than 0.01) [59,60]. The sequences that did not match known miRNAs were mapped to the rainbow trout genome [61] to identify potentially novel miRNAs. Novel miRNAs were predicted if the extended sequences (~60 nt in both directions) at the aligned positions have the propensity to form hairpin structures as analyzed using RNAfold program.

The reads for each miRNA (either known or novel) were aligned by Clustal analysis using CLC Genome Workbench (CLC bio, MA). Quantile normalization in Limma package was used to normalize the miRNA reads [62]. The difference in miRNA expression between high quality egg (D1PO) and low quality egg (D14PO) was evaluated by Z-test [63].

### RT-qPCR analysis of miRNA expression

Two μg of DNase-treated RNAs (4 pools from D1PO or D7PO or D14PO eggs) were converted to cDNA using miScript reverse transcriptase mix (Qiagen, Valencia, CA). The cDNA samples were used for RT-qPCR quantification and melting curve analysis of miRNAs using miRNA specific primers (Additional file 1: Table S6) in combination with the miScript universal primer (Qiagen, Valencia, CA). Rainbow trout β-actin and 18S rRNA genes were used as endogenous controls. RT-qPCR was performed in duplicate for each cDNA on a Bio-Rad CFX96 system. The iQ™ SYBR Green Supermix (Bio-Rad, Hercules, CA) was used in 20 μl reaction volumes containing 100 nM of each primer and 5 μl of 1:150 diluted cDNA. Cycling parameters were 95°C for 3 min followed by 40 cycles of 95°C for 10 sec and 50 to 62°C for 1 min. Melting curve analyses were programmed following the amplifications. Standard curves for all miRNAs and the endogenous controls were constructed using a serial dilution of a pooled cDNA sample. For each sample, the quantity of the specific miRNAs and the reference genes was determined from respective standard curves. The mean quantity of the specific miRNAs was then divided by the geometric mean of the 2 reference genes to obtain a normalized value. Mean differences in expression levels were reported as relative fold changes using the lower expression value as a calibrator. Differences in miRNA expression were determined by one-way analysis of variance (ANOVA) using R software.

### Identification of miRNA targets via computational analysis

Two miRNA target prediction algorithms, miRanda (http://www.microrna.org/microrna/home.do) [64] and PITA (http://genie.weizmann.ac.il/pubs/mir07/mir07_exe.html) [65] were used to identify the target genes of the egg quality related miRNAs. Sequences of 15,387 3’UTRs and 14,788 coding regions fetched from rainbow trout transcripts as described previously [45] were used in the analysis. The rainbow trout mitochondrial genome sequence (L29771) was downloaded from the GenBank database [66]. The thresholds of miRanda for candidate target sites were paring score S ≥ 150 and energy score ΔG ≤ −18 kcal/mol, where S is the sum of single-residue-pair match scores over the alignment trace and ΔG is the free energy of duplex formation from a completely dissociated state which was calculated using the Vienna package [64]. The score ΔΔG ≤ −15.0 was used for PITA [67].

### Gene ontology analysis

The online software blast2go (http://www.blast2go.com) was used to analyze the target genes of differentially expressed miRNAs. Enrichment of the gene ontology (GO) terms was tested using hypergeometric function as described earlier [68], and the 96,546 rainbow trout unique transcript sequences (http://www.animalgenome.org/repository/aquaculture) were used as background. Adjusted p-values by Benjamini and Hochberg false discovery rate (FDR) method were used to determine significance of GO enrichment.

## Additional files

Additional file 1: Table S1. Known miRNAs identified from rainbow trout eggs of different quality. **Table S2.** Novel miRNAs predicted from rainbow trout eggs of different quality. **Table S3.** Differentially expressed miRNAs in D1PO vs. D14PO eggs (fold change greater than 3). **Table S4.** The predicted target genes of 6 differentially expressed miRNAs between D1PO and D14PO eggs validated by RT-qPCR analysis. **Table S5.** Target genes associated with siginificamtly enriched GO terms. Table S6 Primers used to validate differentially expressed miRNAs. Additional file 2: Figure S1. Melt peak charts of 7 miRNAs showing specific amplifications in RT-qPCR analysis. β-actin and 18S rRNA are endogenous control genes.

## Abbreviations

miRNA: microRNA
RT-qPCR: Real-time quantitative PCR
D1PO: One day post ovulation
ANOVA: Analysis of variance
GO: Gene ontology
3′UTR: 3′untranslated region
