# Supplementary figures and images for: MicroRNA expression profiles from eggs of different qualities associated with post-ovulatory ageing in rainbow trout (Oncorhynchus mykiss)
Source: BMC Genomics. 2015 Mar 17;16(1):201. doi: 10.1186/s12864-015-1400-0 (PMC4374207; doi:10.1186/s12864-015-1400-0)

## Slide 1
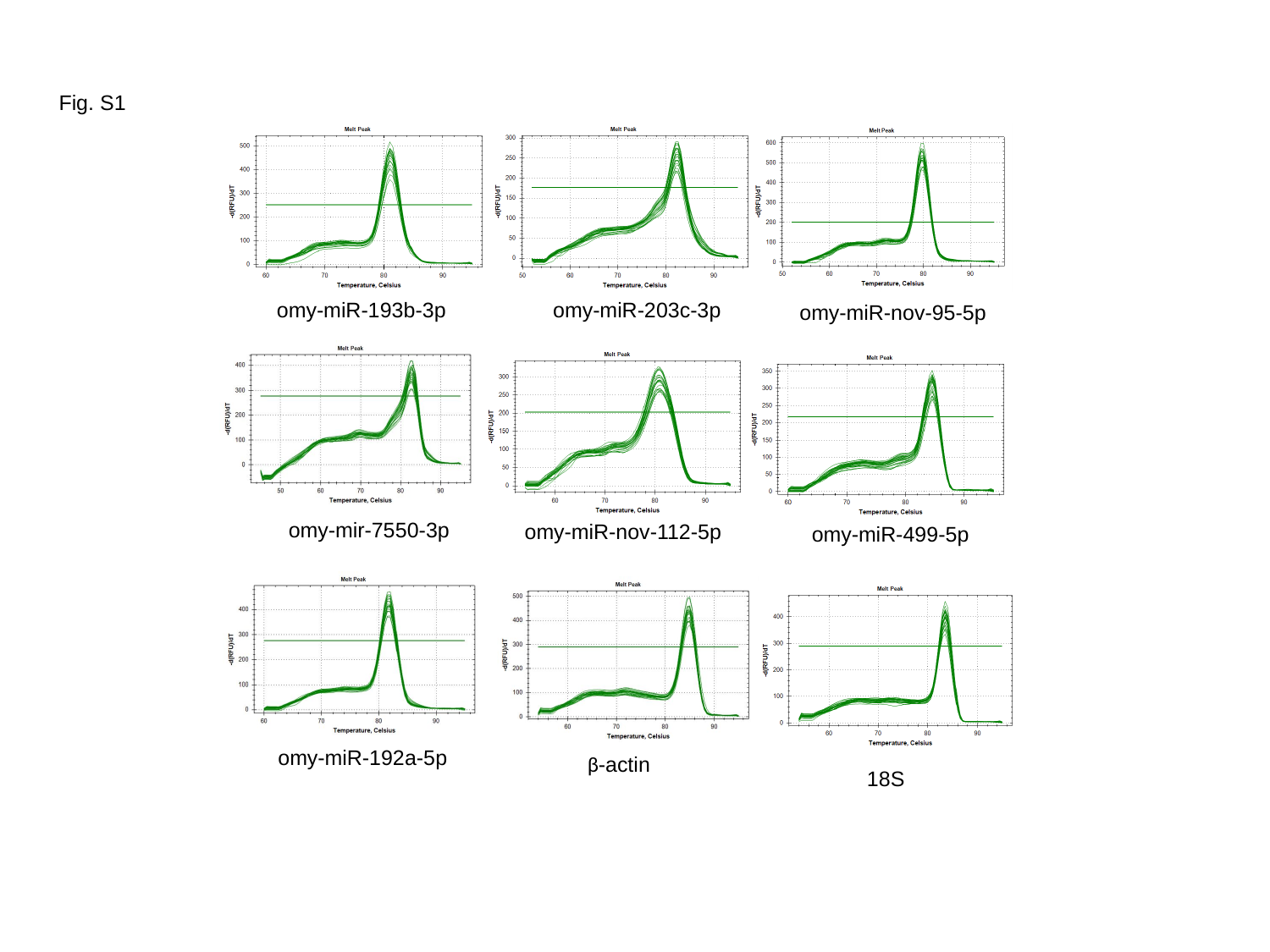

Fig. S1
omy-miR-193b-3p
omy-miR-203c-3p
omy-miR-nov-95-5p
omy-mir-7550-3p
omy-miR-nov-112-5p
omy-miR-499-5p
omy-miR-192a-5p
β-actin
18S

Supplement: Additional file 2: Figure S1. — Melt peak charts of 7 miRNAs showing specific amplifications in RT-qPCR analysis. β-actin and 18S rRNA are endogenous control genes. [file 12864_2015_1400_MOESM2_ESM.pptx]
